# Supplementary material for: A game-theoretic analysis of Wikipedia’s peer production: The interplay between community’s governance and contributors’ interactions
Source: PLoS One. 2023 May 1;18(5):e0281725. doi: 10.1371/journal.pone.0281725 (PMC10150990; doi:10.1371/journal.pone.0281725)
Supplement: S1 File — (PDF) [file pone.0281725.s001.pdf]

Appendix-1: Nash Equilibrium of the Non-Cooperative Game Between the Contributors

Here, we detail the steps in obtaining the optimal contributions Re-writing Equation (3) in the main article,

$$\left(\sum_{j=1}^{N} x_j^*\right)^2 - \alpha_i \sum_{\substack{j=1 \\ j\neq i}}^{N} x_j^* = 0, \quad \forall N, \tag{1}$$

where

$$\alpha_i \triangleq \left(\frac{1}{t+1+\frac{L\beta_i - 1}{w_i}}\right). \tag{2}$$

Equation (1) can be written as

$$\left(\mathbf{x}^*\right)^T \mathbf{1}\mathbf{1}^T \mathbf{x}^* \mathbf{1} - \mathbf{D}_\alpha \left(\mathbf{1}\mathbf{1}^T - \mathbf{I}\right) \mathbf{x}^* = \mathbf{0}, \tag{3}$$

where $(.)^T$ represents the transpose of a vector or a matrix, $\mathbf{D}_\alpha$ is the diagonal matrix $\mathbf{diag}\left(\alpha_1, \alpha_2, \cdots, \alpha_N\right)$, $\mathbf{1}$ is the column vector in which all entries are one, $\mathbf{0}$ is the column vector in which all entries are zero and $\mathbf{I}$ is the identity matrix.

It can be easily verified the vectors, $\mathbf{y}_1 = \frac{1}{\sqrt{N}}\mathbf{1}$ and for $j = 2, 3, \cdots, N$, $\mathbf{y}_j = \left[y_{kj}\right]_{1\leq k\leq N}$, where

$$y_{kj} = \begin{cases} -\frac{1}{\sqrt{j(j-1)}} & k < j \\ \frac{j-1}{\sqrt{j(j-1)}} & k = j \\ 0 & k > j, \end{cases} \tag{4}$$

form a set of orthonormal eigen vectors to the matrix, $\mathbf{1}\mathbf{1}^T$. The eigen value corresponding to $\mathbf{y}_1$ is N and those corresponding to $\mathbf{y}_2, \cdots, \mathbf{y}_N$ are 0s. Let

$$\mathbf{P} = \left[\mathbf{y}_1 | \mathbf{y}_2 | \cdots | \mathbf{y}_N\right]. \tag{5}$$

Then, $\mathbf{P}$ is an orthogonal matrix and by orthogonality transformation [82],

$$\mathbf{P}^T \mathbf{1}\mathbf{1}^T \mathbf{P} = \mathbf{D} = \mathrm{diag}\left(N, 0, 0, \cdots, 0\right). \tag{6}$$

Let $\mathbf{z} = \left[\begin{array}{cccccc} z_1 & z_2 & z_3 & \cdots & z_{N-1} & z_N \end{array}\right]^T$. Since the eigen vectors of a matrix form a basis for the $N-$dimensional sub-space [82], the vector, $\mathbf{x}^*$, can be written as $\mathbf{x}^* = \mathbf{P}\mathbf{z}$. In other words, from Equations (4) and (5),

$$x_1^* = \frac{z_1}{\sqrt{N}} - \sum_{j=2}^{N} \frac{z_j}{\sqrt{j(j-1)}}, \tag{7}$$

$$x_k^* = \frac{z_1}{\sqrt{N}} + \frac{(k-1)z_k}{\sqrt{k(k-1)}} - \sum_{j=k+1}^{N} \frac{x_j}{\sqrt{j(j-1)}}, \; 2 \leq k \leq N-1, \tag{8}$$

$$x_N^* = \frac{z_1}{\sqrt{N}} + \frac{(N-1)z_N}{\sqrt{N(N-1)}}. \tag{9}$$

Intuitively, the vector, $\mathbf{z}$ is a linear transformation of the set of variables in the vector, $\mathbf{x}^*$, which enable solving the set of Equations characterized by (3), using an approach similar to that outlined in [104], described by the following steps.

- Using $\mathbf{x}^* = \mathbf{Pz}$ in Eqn. (3) and Equation (6), we obtain

$$\mathbf{z}^T\mathbf{Dz1} - \mathbf{D}_\alpha\left(\mathbf{11}^T - \mathbf{I}\right)\mathbf{Pz} = \mathbf{0}. \tag{10}$$

- The above is a set of non-linear equations in $\mathbf{z}$, in which the kth equation depends on z_1 and z_j, $k \leq j \leq N$. Solving the non-linear equations by backward substitution [82], z_k, $2 \leq k \leq N$ can be written in terms of z_1 as

$$\frac{z_k}{\sqrt{k(k-1)}} = \frac{Nz_1^2}{k(k-1)}\left[\frac{k}{\alpha_k} + \sum_{j=k+1}^{N}\frac{1}{\alpha_j}\right] - \frac{z_1}{\sqrt{N}}\frac{N(N-1)}{k(k-1)}. \tag{11}$$

- Using Equation (11) to replace all z_k's in terms of z_1 in the set of non-linear equations in Eqn. (10), z_1 can be obtained as

$$z_1 = \frac{N-1}{\sqrt{N}}\frac{1}{G}, \tag{12}$$

where

$$G \triangleq \sum_{j=1}^{N}\frac{1}{\alpha_j}. \tag{13}$$

- Combining Equations (11) and (12), for $2 \leq k \leq N$,

$$\frac{z_k}{\sqrt{k(k-1)}} = \frac{(N-1)^2}{k(k-1)}G^{-1}\left[G^{-1}\left(\frac{k}{\alpha_k} + \sum_{j=k+1}^{N}\frac{1}{\alpha_j}\right) - 1\right] \tag{14}$$

- Using Equations (7)-(9) and α_i from Equation (2) in Equation (12) and Equation (14), the unique Nash equilibrium, $\mathbf{x}^*$ can be obtained as given by

$$x_i^* = \frac{\sum_{j=1}^{N}\left(\frac{L\beta_j-1}{w_j}\right) - (N-1)\left(\frac{L\beta_i-1}{w_i}\right) + t + 1}{\left(\sum_{j=1}^{N}\left[\left(\frac{L\beta_j-1}{w_j}\right) + t + 1\right]\right)^2}, \tag{15}$$

which is Equation (4) in Theorem 1 in the main manuscript.

References

104. Anand S, Sengupta S, Chandramouli R. Price bandwidth dynamics for WSPs in heterogeneous wireless networks. Elsevier Physical Communications. 2014;14(12):63-78.
