# Supplementary material for: A game-theoretic analysis of Wikipedia’s peer production: The interplay between community’s governance and contributors’ interactions
Source: PLoS One. 2023 May 1;18(5):e0281725. doi: 10.1371/journal.pone.0281725 (PMC10150990; doi:10.1371/journal.pone.0281725)
Supplement: S2 File — (PDF) [file pone.0281725.s002.pdf]

Appendix-2: Determining the Optimal value of Neutrality Enforcement, t

We now present the detailed analysis to show the fact that the optimal value of t that maximizes the entropy of a page is $t \to \infty$. From Equation

$$\frac{\partial H}{\partial z} = -\sum_{i=1}^{N} \frac{\partial c_i^*}{\partial z}(1 + \ln\, c_i^*). \tag{1}$$

Therefore, the condition $\frac{\partial H}{\partial z} = 0$ yields the condition,

$$\sum_{i=1}^{N} \frac{\partial c_i^*}{\partial t} \ln\, c_i^* = -\sum_{i=1}^{N} \frac{\partial c_i^*}{\partial t} \ln\, c_i^*. \tag{2}$$

From Equation (6) in the main article,

$$\frac{\partial c_i^*}{\partial z} = \frac{(N-1)\left(N\frac{L\beta_i - 1}{w_i} - \sum_{j=1}^{N} \frac{L\beta_j - 1}{w_j}\right)}{\left(Nz + N + \sum_{j=1}^{N} \frac{L\beta_j - 1}{w_j}\right)^2} \tag{3}$$

$$\text{i.e., } \frac{\partial H}{\partial z} = 0 \Rightarrow \sum_{i=1}^{N} \frac{\partial c_i^*}{\partial z} = 0. \tag{4}$$

Putting Equation (4) in Equation (2) and simplifying, the optimal neutrality enforcement, t is obtained as the value of z that solves

$$\sum_{i=1}^{N} \left[\left(\frac{\beta_i}{w_i} - E\left(\frac{\boldsymbol{\beta}}{\mathbf{w}}\right)\right)\right] \ln\, c_i^*(z) = 0. \tag{5}$$

From the observations listed in Section 4.1 in the main article, if $\frac{\beta_i}{w_i} > E\left(\frac{\boldsymbol{\beta}}{\mathbf{w}}\right)$, then $c_i^* = 0$, i.e., $lnc_i^* \to -\infty$. $0 < c_i^* < 1$ when $\frac{\beta_i}{w_i} < E\left(\frac{\boldsymbol{\beta}}{\mathbf{w}}\right)$, i.e., $\ln\, c_i^* < 0$. Therefore, the value of t that satisfies Equation (5) is the value that makes $\ln\, c_i^*$ go to $-\infty$, i.e., make $c_i^* \to 0$, i.e., $t = \infty$, from Equation (6) in the main article. Therefore, the value of t that maximizes $H(t)$ is $t \to \infty$.
