# Supplementary material for: A game-theoretic analysis of Wikipedia’s peer production: The interplay between community’s governance and contributors’ interactions
Source: PLoS One. 2023 May 1;18(5):e0281725. doi: 10.1371/journal.pone.0281725 (PMC10150990; doi:10.1371/journal.pone.0281725)
Supplement: Attachment — Submitted filename: PONE D-21-20634R2 RebuttalLetter.pdf [file pone.0281725.s006.pdf]

PONE-D-21-20634R2: A Game-Theoretic Analysis of Wikipedia's Peer Production: the Interplay between Community's Governance and Contributors' Interactions

Feedback on our 2nd revision (3rd submission, counting the original)

Once again, we thank the review committee for their continued guidance. Now that reviewer 1 has no additional comments, we focused our attention on the important points that were raised by Reviewer 2. We believe that we were able to address the few remaining issues, and hope that the paper is now ready for publication at PLOS ONE.

Reviewer #2:

The authors have addressed almost all of my prior comments in this revision. I want to commend the authors in their efforts to update the model, as well as to re-run all of the empirical analyses. I am generally happy about the state of the paper at this point, and just have a few minor edits plus some editorial suggestions. I hope the authors find them useful. Given that these suggestions are not fundamental to the paper, I recommend a minor revision at this point. Below are my detailed comments.

> We thank the reviewer for the detailed reading of the paper and their constructive feedback. The reviewer's additional comments helped improve the paper's quality, and we are grateful for that.

Does the quality (sum of all x_i) need a multiplier so that the relative magnitude of quality and the fractional ownership can be better accounted for? I am worried that the way the utility function is modeled in (2), the quality may have an outsized impact on the overall quality compared to the fractional ownership, given that perhaps the magnitude of the argument for quality is going to be significantly larger than the fractional ownership argument. I am not sure if this impacts anything, and perhaps it doesn't, but just a quick confirmation that this is not driving the results would help.

> We thank the reviewer for raising this important issue. We have tried to address it both empirically and analytically.
>
> We begin the description of the empirical analysis by recalling that our model specifies two types of contributors – the vast majority of contributors with a competitive orientation (w_i close to 1) and the other with a cooperative orientation (w_i close to 0) – each deriving distinct benefit from participating in Wikipedia's collaborative authoring process. Specifically, the "benefit" side of our utility function includes two components, the competitive benefit (i.e. fractional ownership) is weighted by w_i and the cooperative benefit (i.e. high-quality article) is weighted by 1- w_i. In a sense, these weights work as a scaling factor.
>
> Empirically, we find that these weights keep the values for these two components comparable. For example, for curators with a competitive orientation whose fractional ownership was more than 0.5, the net utility was of the order of 1.2. That is, although the cooperative benefit (Σx_i) is increasing the utility function, it is *not* out-sizing the competitive benefit (i.e. fractional ownership) by orders of magnitude. For cooperative contributors, on the other hand, the cooperative benefit does dominate the competitive benefit, but these cooperative contributors do not own much content (as our results have already shown before), and thus this imbalance between the two benefit components does not affect the pattern of results.
>
> We also performed an additional analysis by including a scaling factor $\theta \ll 1$. Our findings show

that this did change the analytical results. Whereas there was a change in the Nash equilibrium for content ownership (namely, $\Sigma(L\beta_i-1)$ was replaced by $\Sigma(L\beta_i-\theta)$), we found that this change had very little effect on our numerical results, since $L\beta_i$ dominated in the final expression. We also note that our asymptotic results hold even when including this scaling factor.

We considered whether to include the scaling factor in the revised manuscript, but opted to leave it out, given that it complicates the model, yet adds no additional insight. After all, modelling should strive for parsimony.

That being said, if R2 thinks that it is better to include the scaling factor in our conceptualization, we are happy to do so in the next iteration.

The asymptotic results in lines 574-587 states that the result are in “the presence of Wikipedia's governance to ensure neutrality”, however, the result from Theorem 4 seems to hold irrespective of t. The authors need to clarify that this is just because of the costs and benefits through the utility function other than t that those users with low edit sizes and high competitiveness end up owning more share. It might be nice to add a bit of intuition here based on not just the asymptotic case, but also the general case in Theorem 3 on why this happens.

We agree with the reviewer that the asymptotic result (Theorem 4) does not depend on t, and we are grateful to the reviewer for pushing us to clarify this issue.

First, to place the comment in context, we note that in this part of the Results section we present our findings and only briefly allude to the implications. Later, in the Discussion section, we interpret the study’s findings in length and elaborate on their implications.

As mentioned by the reviewer, the content ownership (Equation (6) from theorem 3) does depend on the governance level, t. Since Equation (6) is too complex to intuitively explain, we chose to provide the intuitive reasoning for the asymptotic result (i.e. when the number of contributors becomes very large), as presented in Theorem 4.
Hence, when describing the asymptotic result, we removed the sentence that stated this result is “in the presence of governance”.

Nonetheless, we stress that the fact that t falls out in the derivation that yields the equation in Theorem 4 (Equation (8)) does *not* mean that governance levels do not affect the general pattern of results. As we demonstrate later in the paper, governance levels do in fact influence the cooperation/competition dynamics (e.g. excessive levels of governance bring the process to halt).

In the Discussion section (specifically, Section 6, line 856 in the revised manuscript) we discuss the way in which governance policies that enforce neutrality influence our empirical findings (namely, curators with competitive orientation ending up owning more content).

I think I didn’t properly explain what I meant before when I asked the authors to take a look at the plot that has w and \beta on two dimensions. I was asking for the plot from the game-theoretic model on these two. Basically, for a given parameter set, a plot can be drawn to show for example that those users with w/\beta < E[W/\Beta] own a non-significant portion of the content, whereas those with w/\beta > E[W/\Beta] do not own any of the content per Theorem 4 (or perhaps find a way to discuss the findings in Theorem 3?).

The revised manuscript includes a newly-added line that specifies $E(\beta/w)$ on the plots, as shown below. Now it is more clear to see that only users with a ration that is larger than the group’s average end up owning content.

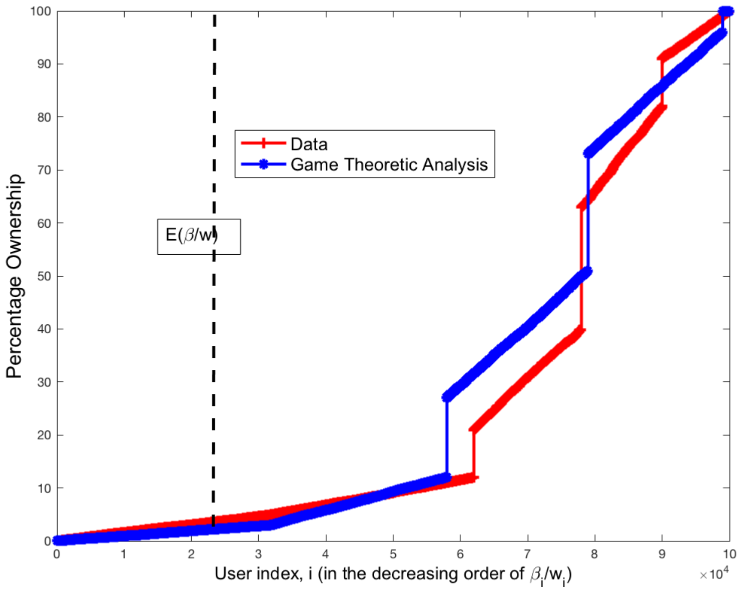

Figure 1: All Years

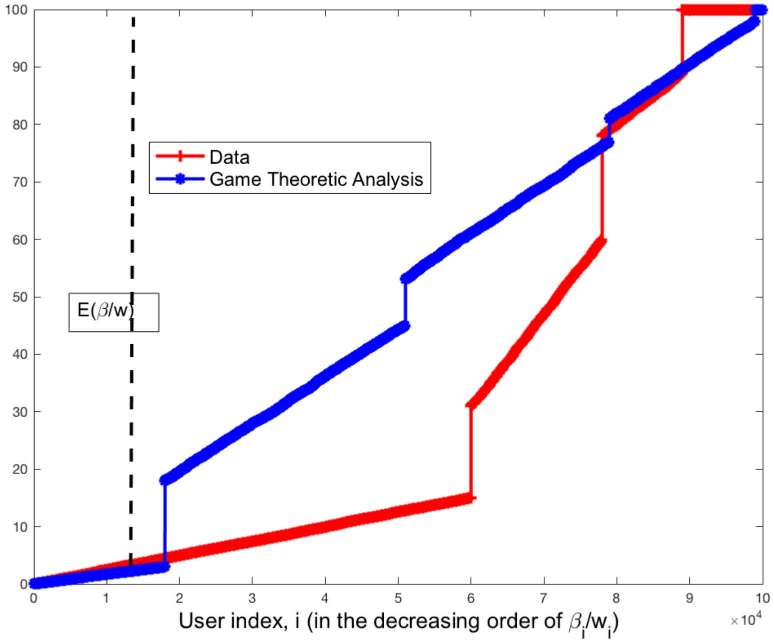

Figure 2: First Year

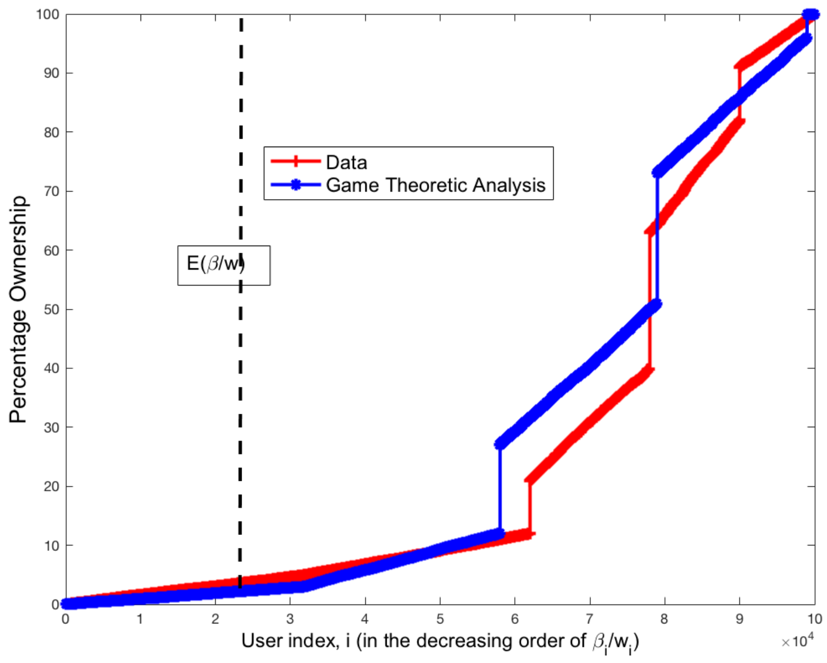

Figure 3: Second Year

In the literature review section, it might be useful to further clarify the contribution to each of the three sub-fields and quickly compare the stated literature to the current paper on the aspects that are important and relevant.

In the literature review section, we review three relevant stream of research, each in a distinct subsection:

a. Cooperation and Competition in Wikipedia's Content Production
b. Contributors' Activity Profiles: Creators and Curators
c. Governance of Wikipedia's Content Production: Neutral Point of View (NPOV) Policy

Our intention for that section is to describe the state of the art, with a focus on the angles most pertinent to our study's research question.

In the Discussion section, once the study's findings have been presented, we come back to the different streams within the literature which were reviewed earlier, and discuss how our findings inform and contribute to each of these streams. Whereas there is not a 1:1 mapping between the structure of the Literature Review and the Discussion sections, we did strive to make linkages. In particular:

- The 3rd paragraph (with starts with "We note that prior studies have discussed individual contributors' attempts to influence articles' contents …") discusses our contribution to the literature on the competition in Wikipedia's content production (i.e. the attempt to "own" article portions).
- The paragraph that follows (beginning with "The finding from the non-cooperative game that a competitive orientation yields …" discuss our paper's contribution to the literature on contributors' roles, specifically creators and curators.
- The next paragraph (which begins with "A second powerful result of this study is in demonstrating that") discusses our contribution to the study of Wikipedia's governance,

specifically in highlighting some of the negative side-effects that are associated with excessive levels of governance.

When introduction \beta$_i$, clarify the continuum for it: does high \beta mean creator or curator? This is formally clarified much later on line 567.

Once again, the reviewer has raised a good point. We have kept the explanation of β being a continuum, both in the introduction and in the analysis section.

The y-axis on Figure 4 goes from 0 to 10^0, perhaps better to note it as 0 to 1?

This is an artifact of Matlab. When we plot on linear scale the variation is not visible. Hence we used a semi-log scale and therefore, the label appears as 10^0.

Research question may be better provided not in a section of its own, though this is subjective and I let the authors decide whether to change anything about this.

We have considered integrating the section on research question into the literature review chapter (i.e. making it Section 2.4), but we think that it is more appropriate to leave it as a separate (short) section.

Writing: I found a couple of issues, you may want to have another proof-reading before the final version

- On line 430, the "a" before "either" should be removed and there is an extra space after the "(".
- On line 574, it should be "who ARE creators of content".

We now have carefully proofread the paper and corrected all grammatical and typographical errors.
