# Supplementary material for: A game-theoretic analysis of Wikipedia’s peer production: The interplay between community’s governance and contributors’ interactions
Source: PLoS One. 2023 May 1;18(5):e0281725. doi: 10.1371/journal.pone.0281725 (PMC10150990; doi:10.1371/journal.pone.0281725)
Supplement: Attachment — Submitted filename: SA-OA-NM-ON-PLOS-One-Final-Rebuttal Letter.docx [file pone.0281725.s004.docx]

**PONE-D-21-20634: A Game-Theoretic Analysis of Wikipedia’s Peer-Production: the Interplay between Community’s Governance and Contributors’ Interactions**

**Rebuttal Letter**

Dear Prof. Francisco Alvarez Gonzalez, Academic Editor, *PLOS One,*

We would like to thank the Academic Editor and reviewers for their valuable feedback and their insightful comments. We have worked hard to address the important concerns raised by the review team. As a result, the paper has improved along four key dimensions:

- The most substantial change in this revision was the incorporation of *cooperation* between contributors in our conceptualization, in addition to our treatment of the *competitive* aspects of peer-production. As a result, the paper’s narrative and write-up have changed considerably, particularly in the front-end. Modelling wise, this entailed adding cost and benefit components to the utility function. In particular, the modified utility function now includes the benefits associated with the collaborative production of comprehensive high-quality articles. This substantial enhancement of our conceptualization required that we defined a new variable, w_i_, indicating the extent to which one is cooperative/competitive. All cost and benefit components in our new model are a function of the contributor’s cooperative/competitive orientation. For example, the competitive benefit of “owing” article portions is a function of w_i_, whereas the cooperative benefit of producing a comprehensive article is a function of 1- w_i_.
- Conceptualization of creators/curators and modelling of β_i_. The revised manuscript provides a better support for our conceptualization of contributors’ activity profiles. We better support our argument that creators typically make larger-size edits, when compared to curators, and provide support for our argument that these activity profiles are stable over time (Arazy et al. 2016). We define β_i_ as the contributor’s position on the creator-curator continuum (0 <β_i_<1). Consequently, the effort is making one’s average size edit is defined as a linear function of β_i_, i.e. Lβ_i_. We further define the number of revisions a contributor makes to the focal article is defined as x_i_, and thus the cumulative effort a contributor exerts in editing an article is Lβ_i_x_i_. We conceptualize contributors’ characteristics, in terms of both their creator/curator activity profile (β_i_) and their cooperative/competitive orientation (w_i_) as static traits (within the yearly temporal brackets that are used in our empirical analysis), and the quantity of one’s activity (x_i_) as the contributor’s choice variable. It is important to note that the two traits – one’s cooperative/competitive orientation and her creator/curator activity profile – are largely independent from each other (Arazy et al. 2019). For example, a core member of the community (i.e. having a cooperative orientation) may take the role of a creator or alternatively of a curator. Hence, our model treats β_i_ and w_i_ as independent variables.
- Novel findings. The results of the enhanced model reveal that the factor that determines who end up owning content is the contributor’s characteristics, or more specifically the ratio between one’s position the creator-curator continuum and the contributor’s cooperative/competitive orientation (β_i_ /w_i_). When this ratio is smaller than the group’s average, the contributor maintains ownership over portions of the article. Namely, under the governance mechanisms, the fractional content that is eventually owned by a contributor is higher for curators (i.e. with a typical small-size edit) that have a competitive orientation. In essence, over time, those with a cooperative orientation and those who regularly make large-size edits (i.e. creators) loose ownership of the article.
- As a consequence of these revisions to our conceptualization, we made significant enhancements to our empirical analyses. First, we operationalized contributors’ cooperative/competitive orientation and identify a measurement proxy for this orientation, namely whether one is a core member of the community (i.e. has a more cooperative orientation) or a peripheral member (less cooperative). Second, given the newly added variable and the new results of the Nash equilibrium, we re-did the entire empirical analysis, including all the robustness checks. We note that although the overall patterns described in the new figures (namely, Figure 3a-c) largely resemble the figures from our original manuscript, the X axis has changes (now, β_i_ /w_i_) and so have contributors’ positions on that axis.

We also made many other changes to the manuscript, per the reviewers’ comments. Please also note that to reflect these revisions, we have changed the paper’s title to “A Game-Theoretic Analysis of Wikipedia’s Peer-Production: the Interplay between Community’s Governance and Contributors’ Interactions” (the original manuscript’s title was “A game-theoretic analysis of competitive editing in Wikipedia: Contributors' effort to influence articles and the community's attempt to ensure neutrality”).

Below, we expand on these issues and respond to each of the editor’s and reviewers’ comments point-by-point.

In addition to the rebuttal letter and revised manuscript, we have also submitted a file that tracks the changes we have made to the manuscript. Given that the changes run deep and are manifested throughout the manuscript, it was not possibly to track changes on a word-by-word basis, Instead, we have highlighted the key places where the text changed considerably.

We thank the review team for helping us improve the paper and appreciate their constructive comments. We also thank you for allowing the extension of the submission deadline; a member of our team suffered from a serious medical issue, which hampered our ability to work on the revision. We are happy to have completed the revision and rebuttal letter in time.

We believe the current manuscript is stronger both theoretically and empirically, and hope that our paper is now closer to being ready for publication at *PLOS One*.

**Academic Editor (AE)**

**AE.0** Thank you for submitting your manuscript to PLOS ONE. After careful consideration, we feel that it has merit but does not fully meet PLOS ONE’s publication criteria as it currently stands. Therefore, we invite you to submit a revised version of the manuscript that addresses the points raised during the review process.

Response: Thank you for affording us the opportunity to revise and resubmit the paper.

**AE.1** At this point two referees have reported. One of them suggests rejection, while the other suggests a major revision. I suggest you to revise and re-submit your manuscript in line with the comments of this latter referee. I want to make clear that my suggestion does not convey any commitment of future acceptance. The real contribution of the current version of the paper seems to be vague and unclear even for the most pro-acceptance referee, which makes me be very cautious at this stage.

Response: Thank you for this guidance. Per the AE’s suggestion, we have focused the revision on the comments made by R2. Nonetheless, we have also worked hard to address all the concerns that were raised by R1. In fact, we found that many of the reviewers’ comments were aligned, such the changed we made often tackled concerns that were raised by both R1 and R2. We believe that the revisions address the concerns of both reviewers.

**Reviewer 1 (R1)**

**R1.0** The paper uses a game-theoretic model to analyze editing behavior on Wikipedia and in particular, how competition for ownership of an article affects the incentives of individual users to contribute. The paper also analyzes how the Wikipedia’s governance structure influences editing behavior by altering the contribution incentives. The key prediction that emerges from the analysis is that users that make longer edits are less likely to have final ownership over an article (this pattern is verified empirically) and that stricter rules of enforcement on Wikipedia can discourage edits from users that tend to make longer edits (this prediction comes out of the model but is not empirically verified).

Editing dynamics on Wikipedia and related platforms are interesting and understanding how governance rules impact editing is important. Having said that, I have my reservations about the current draft for a few reasons.

Response: Thank you for this synopsis of the paper. We have worked to address all of the reviewer’s comments, as detailed below.

**R1.0.1** I do think the prediction about edit length and ownership is driven by an assumption that is baked into the model setup and that I find hard to justify.

Response: If we understand this point correctly, R1 suggests that the original model’s articulation of the cost function (i.e. edit size) drove the results re content ownership (namely, those exerting more effort in making larger edits will end up owing less content). Based on the reviewers’ comments, we have fundamentally revised our conceptualization and game-theoretic model, as outlined in the opening of this rebuttal letter.

Namely, we incorporated into our conceptualization cooperation between contributors, in addition to our treatment of the competitive aspects of peer-production. This entailed adding cost and benefit (namely, the benefits associated with the collaborative production of comprehensive high-quality articles) components to the utility function. Hence, we defined a new variable, w_i_, indicating the extent to which one is cooperative/competitive. All cost and benefit components in our new model are a function of the contributor’s cooperative/competitive orientation.

We also refined the conceptualization of creators/curators and the modelling of β_i_. The revised manuscript provides a better support for our conceptualization of contributors’ activity profiles. We define β_i_ as the contributor’s position on the creator-curator continuum (0 <β_i_<1). Consequently, the effort is making one’s average size edit is defined as a linear function of β_i_, i.e. Lβ_i_. We further define the number of revisions a contributor makes to the focal article is defined as x_i_, and thus the cumulative effort a contributor exerts in editing an article is Lβ_i_x_i_. We conceptualize contributors’ characteristics, in terms of both their creator/curator activity profile (β_i_) and their cooperative/competitive orientation (w_i_) as static traits (within the yearly temporal brackets that are used in our empirical analysis), and the quantity of one’s activity (x_i_) as the contributor’s choice variable.

With these enhancements of our model, we find that the factor that determines who end up owning content is the contributor’s characteristics, or more specifically, the ratio between one’s position the creator-curator continuum and the contributor’s cooperative/competitive orientation. When this ratio is smaller than the group’s average, the contributor maintains ownership over portions of the article. Namely, under the governance mechanisms, the fractional content that is eventually owned by a contributor is higher for curators (i.e. with a typical small-size edit) that have a competitive orientation. In essence, over time, those with a cooperative orientation and those who regularly make large-size edits (i.e. creators) loose ownership of the article.

We maintain that with the current articulation of the model, content ownership is not directly driven by edit size.

**R1.0.2** The second prediction regarding the impact of enforcement I believe is largely driven by the focus solely on competition rather than collaborative elements of the editing process. A study of course always needs to focus on a few important elements. But when thinking about the governance mechanism of a complex system, we might be missing important aspects when just focusing on competition.

Response: This important comment has required us to re-think our conceptualization. Essentially, we had to go back to the drawing board, attempting to account for collaboration, while ensuring that we are able to empirically test the model’s predictions. The revised model includes cooperative aspects in both the cost (coordination cost, f_i_; where the cost of handling governance mechanisms levies a larger tax on users disposed competitively) and utility (the benefit derived from co-producing a comprehensive encyclopedic entry) components. The new conceptualization has also required us to introduce a new variable, w_i_ for the i^th^ contributor, which represents the extent to which a contributor is driven by cooperative/competitive goals. We hope that the revised model captures the essential features of Wikipedia – namely, both cooperation and competition – while remaining succinct.

**R1.1** The one aspect of the model that I found unusual is the distinction between edit size (beta_i) for a given user and edit content x_i for the same user. I don’t quite understand why we need two different concepts here and it seems to me that the key result of the model is driven by this somewhat unusual distinction. The authors show that content x_i is a negative function of beta_i in equilibrium and therefore higher beta_i users will have lower ownership (ownership is determined only by x_i and independent of edit size beta_i). In some way the main prediction of the model follows mechanically from the assumption about edit utility: users with larger edit sizes beta_i have a higher cost of contributing and therefore contribute less in equilibrium and end up with lower ownership. The natural question then seems to be why the users with long edits do not simply convert to making smaller edits (given that their objective is to maximize their ownership). However, the model does not allow for that and instead takes edit size at the user level as given and exogenous.

Response: Thank you for surfacing this issue. In order to clarify things, we have refined some of our constructs’ definitions, namely:

- We distinguish between two prototypical types of contributors – creators (who primarily add new content to an article) and curators (who mostly refine existing content) – and define β_i_ as the contributor’s position on the creator-curator continuum (normalized to be between 0 and 1). We explain that creators (i.e. higher β_i_) are associated with larger edits (on average).
- We make the case that the effort of making an edit is linearly correlated with edit size, thus we define the average effort one exerts in making an average edit as Lβ_i_
- The number of revisions a contributor makes to the focal article is defined as x_i_
- And thus the cumulative effort a contributor exerts in editing an article is Lβ_i_x_i_

As the reviewer has pointed out, we argue that contributors typically assume the role of either a creator or a curator (or a combination of the two; β_i_), but they rarely change this prototypical role (i.e. these patterns are stable within the temporal brackets of one year that are used in our empirical analysis; (Arazy et al. 2017)). When selecting their pattern of participation and acting as creators or curators, contributors try to satisfy both cooperative (producing comprehensive high-quality articles) and competitive goals (“owning” portions of the article and thus shaping the viewpoint that is reflected in that article). Some contributors tend to be more cooperative and others more competitive (we now model a contributor’s cooperative/competitive orientation as w_i_). Given contributors’ fixed characteristics – namely, their cooperative/competitive orientation and their prototypical activity patterns (creators/creators) - they make choices regarding their levels of participation (x_i_).

With this enhancement to our model, we show that the amount of content owned is now related to the ratio β_i_/w_i_. This implies that the factor that determines who end up owning content is the contributor’s characteristics, or more specifically, the ratio between one’s position the creator-curator continuum (proxied through her average edit size) and the contributor’s cooperative/competitive orientation (core community members are considered more cooperative). When the ratio β_i_/w_i_ is smaller than the group’s average, the contributor maintains ownership over portions of the article. Namely, under the governance mechanisms, the fractional content that is eventually owned by a contributor is higher for curators (i.e. with a typical small-size edit) that have a competitive orientation. In essence, those with a cooperative orientation and those who regularly make large-size edits (i.e. creators) loose ownership of the article.

Hence, the users with larger edits (creators) do not simply convert to making smaller edits (curators) because: (a) there is a tradeoff between costs and benefits, such that whereas creators benefit more from owning larger portions, they also incur higher cost of making their edits; and (b) it has been empirically shown that contributors’ patterns of activity tend to be rather stable.

**R1.2** A related point is that we are taking a static model of competition and comparing it to a dynamic editing process in the data. My sense for why large edits lead to lower ownership is that large edits tend to occur earlier in the life cycle of an article and edit length decreases over time (because contributions are more about polishing up existing content). The later edits by constructions are more likely to survive in the final version of the article.

Response: In order to test for this effect, we performed our analysis independently on each yearly snapshot of articles’ life. We found that once the co-production stabilizes (Year 2 and onward), our model’s predictions match the empirical results (See Fig. 3 in the revised manuscript). That is, looking at each year independently, our empirical results match well the model’s prediction. Thus, we don’t believe that our findings are driven by the timing of when edits are made.

**R1.2** The question of the optimal enforcement mechanism is a very important one, but I am not sure it is easy to answer it without a more complex model of the editing process. If collaborative motives are important for example, then early contributors that make large edits that are later altered might be perfectly happy with this dynamic and in fact might see the curators as providing complementary works to theirs. One finding in this paper is that stronger enforcement will discourage edits from users that make large edits. But this prediction will depend crucially on whether competition for ownership is the primary driving force.

Response: This comment has led us to revise our model. Our revised conceptualization introduces cooperative goals (in addition to accounting for competition) and costs. Furthermore, in our current model, only self-interested actions (i.e. aimed at increasing one’s content ownership) are taxed, whereas as cooperative contributions are not affected by the “tax” of dealing with the community’s attempt to ensure neutrality.

Even with this modification, we still find that the governance should be curtailed to a maximum limit, beyond which it discourages contributors from making contributions to an article, bringing the co-production process to a halt. For additional details, please refer to our response to R2.9 and to Figure 4 in this rebuttal letter.

**Reviewer #2 (R2)**

**R2.0** This paper provides an analytical model and an empirical analysis to explain the competition between contributors for ownership in crowd-sourced content (as in Wikipedia) and how Wikipedia’s governance of NPOV impacts this. The paper studies an important and interesting topic, and provides intriguing insights on these topics. The combination of the analytical and empirical studies is difficult, but authors make a case for it in this paper. The paper is also generally well written and provides a lot of context and justification for most of the concepts used.

I have a few suggestions for the authors to improve the paper further.

Response: Thank you for the constructive feedback.

**R2.0.1** First, I think the authors can make their contributions a bit more clear. Even though discussions are made about the different parts, it would be useful to see “what is the point of the paper” somewhere early on, including why a combination of analytical and empirical analysis is appropriate. There should also be a case for why this is interesting, why it matters, and why should the readers care about this. Perhaps some potential managerial results would be useful.

Response: We have revised the paper to better motivate the research, explaining why our methodological approach is appropriate, and more clearly elucidating the study’s contributions. In addition, we have now added a paragraph on the managerial implications of our findings.

**R2.0.2** Second, some of the assumptions and stated results from the analytical model seem out of place. For example, the model doesn’t really capture heterogeneous \betas, so I am not sure if the drawn result about creators and curators is valid.

Response: In the revised model β represents the contributor’s position on the creator-curator continuum (0<β<1). Thus, βs are actually heterogeneous, such that each contributor has a distinct position, β_i_.

We also analyzed this empirically. Figures 1 -5 below demonstrate the distributions of β_i_ across all the 864 articles in our sample, each figure for a different temporal snapshot. We find that the likelihood of a contributor being a curator is larger than that of being a creator (i.e. for most contributors, the average edit size is small), as illustrated by the exponential nature of the Probability Density Function (PDF). We performed this analysis for all the articles for distinct yearly snapshots; see Figures 1a-c. From the figures, it is observed that the overall pattern of β_i_ distribution is quite similar across Years 1-3. Figure 2 verifies the exponential distribution of the normalized edit size, β_i_. We note that we also repeated this analysis of β_i_ distribution for individual articles and have found similar results.

|  |  |  |
| --- | --- | --- |
| Probability Density Function of the normalized edit size, β_i_ for Year 1 in articles’ life | Probability Density Function of the normalized edit size, β_i_ for Year 2 in articles’ life | Probability Density Function of the normalized edit size, β_i_ for Year 3 in articles’ life |

Figure 2: Quantile plot for verifying the exponential distribution of the normalized edit size, β_i_

**R2.0.3** Third, the empirical study could improve by some robustness checks, as well as more statistical analysis. Other than these, some of the concepts could be better explained and some assumptions could be better justified. Below I provide my detailed feedback, which I hope can help authors take the paper to the next step in the process, and wish them good luck.

Response: We thank the reviewer for pushing us to rethink our modelling assumptions. We have worked hard to address the various concerns that were raised by the reviewer, making significant modifications to the manuscript. We believe that as a result the paper has improved substantially. In particular, we have clarified concepts’ definitions, better justified our assumptions and performed additional analyses. Please see details below.

Major Comments

**R2.1** Is NPOV the same as equal contribution? Does Wikipedia generally prefer many contributors to fewer, thereby motivating smaller edits? Authors state without enough evidence that often the reason for a lack of neutrality is that one contributor takes substantial ownership. It seems to me that having a NPOV is not the same as having many contributors for an article: one contributor may be able to provide a NPOV on a subject. Therefore, it seems to me that the model should capture these different concepts in a more nuanced way. In other words, the governance does not directly impact the number of contributors, but this is more of an indirect impact, as NPOV may require a more diverse set of contributors. I understand that the analytical model cannot capture the whole reality, however, these two concepts are focal to the analysis. Alternatively, authors can provide additional evidence that shows a direct connection between the two.

Response: The reviewer is making an important point. In principle, an even distribution of activity does not necessarily entail an article having a balanced viewpoint (NPOV). As R2 suggests, it is possible that a single contributor would dominate the discussion, but this contributor would present both sides of an issue, and thus the article would reflect a NPOV.

However, in reality, and given a large enough sample, the set of contributors working on an article would sample a range of viewpoints. The more one contributor (or a subset of the contributors) is able to dominate the discussion (i.e. taking ownership of large portions of the article), the more likely the article is to reflect a single side. The literature has empirically demonstrated that when making edits to Wikipedia articles, contributors tend to “pull” the article towards their personal viewpoints (in addition to working to make the article informative) (Arazy et al. 2020). Hence, our model’s objective function of ensuring an equal ownership (i.e. maximizing entropy) is likely to also ensure a neutral article. The revised paper better explains the linkage between balanced contribution and NPOV.

**R2.2** Another concept related to the point above is that different contributors may have similar POVs, thereby, even as the number of contributors increases, if they all have similar ideas, then NPOV would not be achieved.

Response: Yes; it is possible that all contributors working on a particular article would have the same one-sided position, and thus the article would be biased even if the distribution of activity is uniform. But as we explained above, when considering a sufficiently large sample, the set of contributors working on an article would present diverging viewpoints, and thus a balanced distribution of ownership is likely to result in a NPOV.

**R2.3** Do contributors really try to maximize their ownership? Or are they trying to maximize the informativeness of the articles? Is this concept more prevalent in articles which are amenable to different points of view, for example in case of more controversial articles? Perhaps some analysis of the data on different types of articles (controversial vs non- controversial) would provide additional justification about the competition on ownership.

Response: Again, the reviewer is touching on a key issue. Contributors may either have a cooperative or competitive orientations, as reflected in our revised conceptualization (this orientation captured by the newly-introduced variable w_i_). Whereas the goal of competitive contributors is to take ownership of the article (as represented in our original model), cooperative contributors are interested in collaboratively producing a high-quality article (i.e. maximizing the article’s informativeness); the latter dimension has been now added to the revised manuscript, as we explain in the opening of this rebuttal letter.

The quality of an information good, such as a Wikipedia article, is a multi-dimensional construct, and for an article to be of high-quality it needs to be comprehensive, factually accurate, timely, to mention just a few of the salient dimensions (Arazy and Kopak 2011; Wang and Strong 1996). For simplicity, and in order to allow the measurement of this construct so as to afford the empirical analysis, we focus here on one key dimension of information quality: comprehensiveness (Arazy and Nov 2010). Hence, our revised utility function includes both the cooperative benefit (i.e. producing a comprehensive article, modelled as the sum of all contributors’ “owned” content) and a cost (the cooperative cost, f_i_, is now a function of one’s cooperativeness, i.e. f_i_(1-w_i_).

This is explained in the revised manuscript.

**R2.4** Do the contributors need to put effort in coordination and governance work and sit on committees for Wikipedia? This is somewhat at odds with the assumption that policies don’t change over time. Related to this, from a modeling point of view, L is unnecessary, as it is always multiplied by \beta (the new \beta is L \beta). Also, t could be included in \beta, therefore it is also unnecessary. Basically, there is a fixed cost and a variable cost to editing, and only the total cost is important, as the individual parts do not act differently from one another. Additionally, it is not clear why the contributor incurs the fixed cost for every edit they do? Wouldn’t they become familiar with the platform at some point and thereby not concur the fixed cost? The authors don’t really provide much justification or example for this type of cost.

Response: Wikipedia policies and rules only slowly change (Forte et al. 2009), and could be seen as stable for the yearly temporal brackets that are used in our analysis. When we talk about coordination and governance work we refer primarily to discussions that take place on articles TalkPages and other communication channels (Kane 2011), including the *enforcement* of Wikipedia’s policies and rules. Each article in Wikipedia is accompanied with a discussion forum in a form of a wiki page: a TalkPage. Much research has investigated coordination and policy enforcement that goes on TalkPages and other auxiliary pages (Arazy et al. 2011; Forte et al. 2012; Kiesler et al. 2012; Kittur and Kraut 2008; Kittur and Kraut 2010; Kittur et al. 2007; Zhu et al. 2012). We assume that only cooperative contributors take part in this effort, as reflected in our revised model: coordination cost is f_i_(1-w_i_) (see our response to R2.3 above).

The reviewer is correct that in our original model, some of the variables seem redundant. However, this has been fixed in the revised manuscript. Namely, the constant L is required, given that we model the effort of editing to be a linear function of one’s position on the creator-curator continuum, Lβ_i_. Please refer to our response to R1.1. Conceptually, the distinction is important, although mathematically we could have eliminated the constant L (it falls off in the mathematical derivation).

The variable representing the “tax” of handling governance, t, cannot be conflated onto β_i_, as this tax is applicable for only competitively-disposed contributors. Please see Equation 2.

Lastly, the fixed cost is for the coordination effort that takes place on auxiliary pages, f_i_. It is different for every user, but independent of the activity of co-producing the encyclopedic article. The cost is applicable for cooperative contributors, and thus modelled as f_i_(1-w_i_). We note that this variable is of no consequence to our results. As the reviewer has pointed out, our model does not account for learning effects; we allude to this when discussing directions for future work.

**R2.5** Authors mention quadratic cost, is it possible to get any results with a quadratic function? Such a function makes a lot more sense: it is easy to add a few sentences to an article, but it gets prohibitively hard as they keep editing and adding to an article. (Though I can see the model getting stuck with such a structure.)

Response: We mention in passing that “in general, a convex function of xi would be the most comprehensive model for the cost incurred by each contributor, to own xi amount of content” and further state that “We consider a linear cost model, which is a special case of a convex cost function (Luenberger, 1984)”. We explain that “The complexity of the analysis is very high for general convex cost models and is out of scope of our research“.

When discussing future research direction, we now mention the possibility of a more complex cost function.

**R2.6** It may be better to provide the results in a more formal way, e.g. in a lemma or proposition.

Response: The reviewer raises an interesting point. Typically, we would use a Lemma or a proposition if they lead to an algorithm or if the result of the Lemma or proposition is used to prove other complex results. In our analysis, the results of the game theoretic analysis (validated against empirical data) is used to develop intuitive insights, but does not lead to an algorithm or does not give rise to more complex theorems. The main results – (a) the limit on governance and (b) competitive curators ending up owning content - are directly from the solution of the non-cooperative game. Also, the finding that only contributors whose β_i_/w_i_ are less than the average of the group end up owning content is an asymptotic result (when the number of contributors become arbitrarily large) and hence, we do not present it as a Lemma or a proposition.

**R2.7** Given the topic, heterogeneous user abilities (incurring different costs for editing) seems critical? I understand that this may not be possible to solve for the case of an arbitrary number of contributors, but perhaps the robustness can be shown with a few contributors that are heterogeneous? Next item also considers this issue.

Response: We actually do model heterogeneity in the contributor population, and this heterogeneity is captured in our empirical analysis. Namely, contributors are characterized based on two important factors: their activity profile (i.e. their position on the creator-curator continuum, β_i_; proxied through the average size of their edits) and their cooperative/competitive orientation (proxied through their proximity to the community’s core).

We have presented the exponential distribution of β_i_ in our response to R2.1; please see Figure 1a-c above.

We have also empirically analyzed the distribution of contributors’ cooperative-competitive orientation, w_i_. We find that in our data set w_i_’s are distributed such that 40% of the contributors have a strong competitive orientation (w_i_=0.9), for 33% of contributors w_i_=0.6, 22% have w_i_=0.4, and 5% of the contributors are highly cooperative (w_i_=0.1). This result is inline with what the literature of online communities tells us, i.e. only a small portion of the members belong to the community’s core.

We also analyzed the distribution of contributors’ fractional ownership, as presented in Figure 3 below. We begin with the theoretical Probability Density Function (PDF) fit and proceed as follows. From Equation (8) in the revised manuscript, we attempted to determine a PDF for content ownership c_i_. While the analytical tractability of such an expression is extremely complex, we found that the function $f\left( t \right)=(N-1){(1-t)}^{N-2}$ (where N is the number of authors in the article) fits best the PDF that was obtained from our data. The average content ownership across all articles in our sample, c_i_* , was found to be 8% and the standard deviation was 0.55%.

Figure 3: Fitting a Probability Density Function for the content ownership c_i_* over the entire sample of the 864 articles

Whereas we provided evidence for contributors’ heterogeneity in terms of their creator/curator activity profile, competitive/cooperative orientation, and in the fractional content ownership, the reviewer is correct to point out that our model does not capture heterogeneity in terms of contributors’ knowledge or capabilities. This would add another layer of complexity to our model, and is beyond the scope of the current study. We allude to such an addition when discussing future research direction.

**R2.8** The second result from equation 6 on page 11 is not accurate: given that there is only one type of user in the model (all \betas are the same), one cannot compare the curators and creators at the same time. The model captures either the case where all users are creators or where all contributors are curators, not where both types of users are present. In other words, it compares the case where users on average create more content to the case where on average they create less content. I believe that there is a difference between these two, and authors need to clarify this. Moreover, the connection of the model with the empirical analysis should also be reconsidered, given that the empirical results are based on the difference between contributors’ \betas.

Response: We thank the reviewer for this insight. Based on this comment, we have reformulated the notion of β_i_, such that a contributor may take any position on the creator-curator continuum. Please refer to our response to R1.1. We believe that the linkage between model and empirical analysis is now clear.

**R2.9** In the Wikipedia’s decision in the game, does it have any other goals than NPOV? It may be interesting to see if there are any trade-offs, e.g. in size or number of articles (which may be hindered as governance increases).

Response: As we explain above, our revised conceptualization accounts for both cooperative (co-producing a high-quality article) and competitive (owing article portions) goals, and a contributor’s cooperative/competitive orientation is modeled as w_i_.

From the standpoint of the community, there is only a need to govern to competitive behavior, so as to ensure that articles remain neutral.

The reviewer’s comment has pushed us to further explore tradeoffs. We found that with our new formulation, there is actually a tradeoff between governance (and the tax it imposes on contributors, t) and article comprehensiveness (summation of x_i_). On one hand governance works to ensure neutrality, and thus increases the entropy in contributors’ fractional ownership. On the other hand, governance – because of the tax it imposes on contributors’ activity (linked to competitive behavior) – limits activity levels, and consequently acts to reduce article’s comprehensiveness. Figure 4 below illustrates this tradeoff. As shown, low levels of governance are sufficient to ensure neutrality, such that entropy increases non-linearly with governance levels. Article comprehensiveness is less affected by governance levels slowly decreases with governance. This implies that only light governance is optimal, as it both ensures article neutrality and maintains article comprehensiveness. The revised manuscript discusses this important insight.

Figure 4: Impact of the level of governance, t, on the article neutrality (i.e. entropy) and comprehensiveness (proxied by the article size). The Entropy is measured from empirical data while the article comprehensiveness is obtained from the game theoretic analysis (Equation (4) in the revised manuscript).

**R2.10** The 50% threshold for owning an article seems somewhat arbitrary, are the results robust to other measures of ownership (if there are any other measures for this in the literature for example)? It would also be nice to see some stats on how much of the articles are actually owned by a contributor (i.e. larger than the threshold).

Response: We are familiar with only two operationalizations of content ownership (Arazy et al. 2010) and (Halfaker et al. 2009). We employed the algorithm from Arazy et al. (2010), where ownership is determined at the sentence-level, when one originated over 50% of the words in a sentence. Although the threshold is somewhat arbitrary, that paper demonstrated that the algorithm’s results of ownership are well-aligned with manual assessments regarding the relative value to the article’s contents that is made by each of the contributors.

Figure 2 above presents the distribution of contributors’ fractional ownership.

**R2.11** Rather than the figures for the empirical part, perhaps authors can statistically test their analytical model hypotheses, e.g. the impact of average article size on ownership?

Response: Per the reviewer’s suggestion, we have now analyzed this relationship. Figure 3 above presents the relationship between governance level, t, and article comprehensiveness (proxied based on article size).

**R2.12** Given the timing effects in the empirical analysis, I am confused about something: isn’t the result simply because those who have already made lots of edits have not much left to say, so obviously they would make smaller edits? Is \beta calculated in the context of only the sample of articles or is it their overall value?

Response: Articles in Wikipedia evolve over a prolonged period (for example, our sample covered a period of 11 years). Over this period, articles may shift focus, as the perception of the topic changes in public opinion (and in the opinion of the Wikipedia community); this is particularly true following a news event. For example, the Wikipedia article on the director Peter Jackson saw a spur of activity after the release of the “Lord of the Rings” trilogy. Nowhere is our model do we assume that the size of edits gets smaller over time, and we find no evidence for this effect in our data (please refer to figures 1a-c). Furthermore, our analyses compare the model’s prediction against empirical data for each yearly brackets independently. Given that edits within each bracket are made more-or-less at a similar age of the article, we do not believe that the timing of edits is a factor that influences our findings.

Regarding R2’s second point, we use a large and representative sample of 864 multi-contributor articles, which employed a double-stratified sampling procedure to ensure variance in terms of both topical category and maturity. The calculation of contributor’s average edit size (L β_i_) is based on this sample.

**R2.13** What are the implications of the findings for Wikipedia? How does this impact the quality of articles, number of articles, etc? It would be nice to discuss some of these implications for Wikipedia and other similar platforms.

Response: We have now added a paragraph discussing the study’s practical implications. An excerpt from this new paragraph reads:

Beyond the theoretical contributions and directions for future work, our study's findings also have important implications for custodians of online production communities.

The most important insight pertains to the levels of governance that are required.

Namely, we find that low levels of governance are sufficient for curbing much of the competition over content ownership, ensuring a relatively even distribution of ownership, while only resulting in a very minor reduction in activity levels, thus having a minimal impact on articles' comprehensiveness. Another important contribution pertains to the importance of peripheral community members that have a less cooperative orientation. In fact, as a consequence of their pursuit of content ownership, their activity levels increase, such that they add contents to the article, making the article more comprehensive and of higher quality. Hence, communities such as Wikipedia should encourage peripheral participation (Lave, 1991), in addition to its efforts to encourage cooperative participants to take on additional functions and roles.

**R2.14** Minor issues

**R2.14.1** There are some issues with the writing:

o “loose ownership of the article to those…” should be “lose…”. This error is made in other parts as well. Loose means not firmly attached, lose means to not have something (e.g. lose ownership)

o “and their resulting content ownership” is repeated twice on page 16.

  Response: Thank you for spotting these errors. This has now been fixed.

**R2.14.2** There are some vague concepts in the beginning of the paper, for example, what is Wikipedia’s refactoring process? It would be great to have someone proof-read so that all concepts are explained fully.

Response: We have made effort to explain all concepts in the paper.

In particular, the term refactoring is used in software development to refer to the improvement of a piece of code’s operation without altering its functionality. We now refrain from using this term, and simply discuss the reorganization of existing text.

References used in the rebuttal letter

Arazy, O., Daxenberger, J., Lifshitz-Assaf, H., Nov, O., and Gurevych, I. "Turbulent Stability of Emergent Roles: The Dualistic Nature of Self-Organizing Knowledge Co-Production," *Information Systems Research* (27:4) 2016, pp 792-812.

Arazy, O., and Kopak, R. "On the measurability of information quality," *Journal of the American Society for Information Science and Technology* (62:1) 2011, pp 89-99.

Arazy, O., Lifshitz-Assaf, H., Nov, O., Daxenberger, J., Balestra, M., and Cheshire, C. "On the How and Why of Temporal Dynamics of Emergent Roles in Wikipedia," 20th Conference on Computer-Supported Cooperative Work and Social Computing (CSCW 2017), Portland, OR, USA, 2017.

Arazy, O., Lifshitz‐Assaf, H., and Balila, A. "Neither a Bazaar nor a cathedral: The interplay between structure and agency in Wikipedia's role system," *Journal of the Association for Information Science and Technology* (70:1) 2019, pp 3-15.

Arazy, O., Lindberg, A., Rezaei, M., and Samorani, M. "The Evolutionary Trajectories of Peer-Produced Artifacts: Group Composition, the Trajectories’ Exploration, and the Quality of Artifacts," *Management Information Systems Quarterly (MISQ)* (44) 2020.

Arazy, O., and Nov, O. "Determinants of Wikipedia quality: the roles of global and local contribution inequality," 2010 ACM conference on Computer supported cooperative work ACM, 2010, pp. 233-236.

Arazy, O., Nov, O., Patterson, R., and Yeo, L. "Information Quality in Wikipedia: The Effects of Group Composition and Task Conflict," *Journal of Management Information Systems* (27:4), apr 2011, pp 71-98.

Arazy, O., Stroulia, E., Ruecker, S., Arias, C., Fiorentino, C., Ganev, V., and Yau, T. "Recognizing contributions in wikis: Authorship categories, algorithms, and visualizations," *Journal of the American Society for Information Science and Technology* (61:6) 2010, pp 1166-1179.

Barnett, M. A., Klassen, M., McMinimy, V., and Schwarz, L. "The role of self-and other-oriented motivation in the organ donation decision," *NA-Advances in Consumer Research Volume 14*) 1987.

Forte, A., Kittur, N., Larco, V., Zhu, H., Bruckman, A., and Kraut, R. E. "Coordination and beyond: social functions of groups in open content production," Proceedings of the ACM 2012 conference on Computer Supported Cooperative Work, ACM, 2012, pp. 417-426.

Forte, A., Larco, V., and Bruckman, A. "Decentralization in Wikipedia Governance," *Journal of Management Information Systems* (26:1) 2009, pp 49-72.

Halfaker, A., Kittur, A., Kraut, R., and Riedl, J. "A jury of your peers: quality, experience and ownership in Wikipedia," Proceedings of the 5th International Symposium on Wikis and Open Collaboration, ACM, 2009, p. 15.

Hemetsberger, A. "Fostering cooperation on the Internet: social exchange processes in innovative virtual consumer communities," *NA-Advances in Consumer Research Volume 29*) 2002.

Kane, G. C. "A multimethod study of information quality in wiki collaboration," *ACM Transactions on Management Information Systems (TMIS)* (2:1) 2011, p 4.

Kiesler, S., Kraut, R., Resnick, P., and Kittur, A. "Regulating behavior in online communities," *Evidence-based social design: Mining the social sciences to build online communities*) 2012, pp 125-178.

Kittur, A., and Kraut, R. "Harnessing the wisdom of crowds in wikipedia: quality through coordination," ACM conference on Computer Supported Cooperative Work ACM, 2008, pp. 37-46.

Kittur, A., and Kraut, R. E. "Beyond Wikipedia: coordination and conflict in online production groups," Proceedings of the 2010 ACM conference on Computer Supported Cooperative Work (CSCW'2010), ACM, 2010, pp. 215-224.

Kittur, A., Suh, B., Pendleton, B. A., and Chi, E. H. "He says, she says: conflict and coordination in Wikipedia," in: *Proceedings of the SIGCHI conference on Human factors in computing systems*, ACM, San Jose, California, USA, 2007, pp. 453-462.

Lave, J., and Wenger, E. *Situated Learning Verfified Peripheral Participation*, Cambridge University press, Cambridge, UK.

Wang, R. Y., and Strong, D. M. "Beyond accuracy: What data quality means to data consumers," *Journal of Management Information Systems* (12:4) 1996, pp 5-33.

Zhu, H., Kraut, R., and Kittur, A. "Organizing without formal organization: group identification, goal setting and social modeling in directing online production," Proceedings of the ACM 2012 conference on Computer Supported Cooperative Work, ACM, 2012, pp. 935-944.
