# Supplementary material for: A game-theoretic analysis of Wikipedia’s peer production: The interplay between community’s governance and contributors’ interactions
Source: PLoS One. 2023 May 1;18(5):e0281725. doi: 10.1371/journal.pone.0281725 (PMC10150990; doi:10.1371/journal.pone.0281725)
Supplement: Attachment — Submitted filename: PONE D-21-20634R1 RebuttalLetter.docx [file pone.0281725.s005.docx]

Manuscript PONE D-21-20634 R1: A Game-Theoretic Analysis of Wikipedia’s Peer Production: the Interplay between Community’s Governance and Contributors’ Interactions

Rebuttal Letter

We would like to thank the Associate Editor and reviewers for their valuable feedback and their insightful comments. We have worked to address the outstanding concerns that were raised by the review team.

The most significant change in this revision is the revised definition of the constructs of β_i_ and of x_i_. This required aligning our empirical measures with these new construct definitions and re-running the empirical analysis. We found that although constructs’ values for individual contributors have changed, the overall pattern of results persists (contributors’ percentage of ownership and their trends), and the empirical results still provide a validation for the predictions of the game-theoretic model.

In addition to the changes that directly address the review team’s comments, we have also made a general “clean up”, removing redundant text, adding clarifications where necessary, renaming some variables in the empirical validation section to appear closer to the ones in the analysis and reorganizing the table of notations.

Below, we expand on these issues and respond to each of the editor’s and reviewers’ comments point-by-point.

Besides the revised manuscript and this rebuttal letter, we have also submitted a file that tracks the changes we have made to the manuscript. Given that the changes are manifested throughout the manuscript, it was not possibly to track changes on a word-by-word basis, Instead, we have highlighted the key places where the text changed considerably.

We thank the review team for helping us improve the paper and appreciate their constructive comments.

We believe the current manuscript is stronger and its contribution is now clear. We hope that our paper is now ready for publication at *PLOS One*.

Associate Editor (AE)

**AE.01**: Thank you for submitting your manuscript to PLOS ONE. After careful consideration, we feel that it has merit but does not fully meet PLOS ONE’s publication criteria as it currently stands. Therefore, we invite you to submit a revised version of the manuscript that addresses the points raised during the review process.

At this point two referees have reported. One of them suggests rejection, while the other suggests a major revision. I suggest you to revise and re-submit your manuscript in line with the comments of this latter referee. I want to make clear that my suggestion does not convey any commitment of future acceptance. The real contribution of the current version of the paper seems to be vague and unclear even for the most pro-acceptance referee, which makes me be very cautious at this stage.

Response:

We thank the Associate Editor for the opportunity to revise and resubmit our manuscript. We have worked to address the concerns that were raised by the review team. As a result of these changes, we believe that paper’s contribution is now clear.

Reviewer 1: No comments

Reviewer 2 (R2)

**R2.01**: I wasn't sure why cost of making an edit depends on the contributors activity profile brta_i. Considering the definition of the fractional ownership in c_i which is based on, x_i, this should be a standardized measure of contribution. For example in equation 1 the fractional content ownership is defined as the relative measure of contributed content over total content, which would be incorrect if the size of different x_i’s are different for different types of contributors. In other words, if a creator writes more in one edit compared to a curator, they should still own more of the article. It does not seem to me that the number of edits should be used to calculate the fractions but instead the amount of contribution. Therefore, I'm not sure if the effort involved in a single edit should depend on the type of the user, beta_i. Should beta_i be represented in how much content the user produces instead? In other words, would higher beta users have higher x_i?

Response:

We thank the reviewer for further pushing us to clarify constructs’ definitions. We have now refined the definitions of β_i_ and of x_i_, as well as aligned the empirical measures with these new construct definitions.

Before we describe the changes, allow us to briefly set the stage –

In online production communities such as Wikipedia, contributors are driven by both collective goals (i.e. the co-production of high quality encyclopedic entries) and personal goals (i.e. “taking ownership” of article elements, in an attempt to align article’s content with one’s viewpoint). Costs are associated with each of these goals.

Contributors may have different orientations, cooperative, competitive, or a combination of the two. **w_i_** represents a contributor's cooperative/competitive orientation (0 = entirely cooperative; 1 = entirely competitive orientation).

We view each Wikipedia article as a distinct product, and the collaborative authoring work of developing that article as a group project.

As mentioned above, to advance personal goals (i.e. competitive orientation), contributors may attempt to take ownership of an article’s contents, or of segments of that article. We use a sentence as the basic content unit, although it may be possible to perform the analysis at different granularities (e.g. a paragraph, sub-section, section).

Generally speaking, there are two ways of taking ownership of a sentence:

a. Creating a new sentence; we treat the originator as the owner
b. Shaping an existing sentence by changing, adding or deleting a few words within a sentence. When the amount of changes performed by a contributor surpasses a certain threshold (in our empirical analysis: 50% of the words are new or modified), the contributor making the changes become the owner of that sentence.

Within a certain setting, people tend to adopt particular patterns of activities: some tend to add new sentences (creators); some mostly shape existing sentences (curators); whereas others exhibit a hybrid pattern. We use β**_i_** to indicate one’s pattern of activity (0 = curator; 1 = creator).

We assume that the effort (and thus the cost) of taking ownership of a sentence is a linear function of the amount of content changed within that sentence, such that creating an entirely new sentence requires more effort when compared to changing a few words within the sentence.

Given a contributor’s cooperative/competitive-orientation (w_i_), her regular pattern of activity (β_i_), and the associated costs and benefits, a contributor has the choice of attempting to own any number of sentences. This decision variable, **x_i_**, reflects the contributor’s motivation and availability. Of course, in a coopetitive (cooperative and competitive) setting, the result of one’s action also depends on the actions of others. We use **x_i_*** to represent the number of sentences that the contributor would ideally own, optimizing his benefit/cost ratio.

Our empirical analysis is based on the entire history of article co-production, such that the calculation of both **x_i_** and **β_i_** are based on contributors’ activity over the article’s entire life, up to the study’s date (i.e. over all the iterations/edits made by the contributor).

And now we directly address the reviewer’s concern -

Our calculations of **x_i_** are based on the number of sentences a contributor owns. This reflects the contributor’s activity across all the revisions she has made to the article. Hence, there is no need for our conceptualization to explicitly model the number of revisions made by the contributor.

Consequently, the definition of **β_i_** was revised, and is now based on the typical change that a contributor makes **within a sentence** (previously, it was calculated by considering the number of edits the contributor has made). **β_i_** takes values 0 to 1 in a continuum, i.e., a contributor with value of β**_i_** closer to 0 (typically making very small changes to sentences) has an activity profile resembling a curator, while a contributor with a value of β**_i_** closer to 1 (on average, making large changes to the sentences she edits) is considered a creator of content.

We hope that the distinction between **x_i_** and **β_i_** is now apparent.

Re-running our empirical analyses, we find that although individual contributors’ values have changed, the overall pattern of results persists (contributors’ percentage of ownership and their trends), and the empirical results provide a validation for the predictions of the game-theoretic model. This is true for the analysis for the article’s entire period, as well as for the analysis of yearly snapshots.

**R2.02** Related to the above comment, understanding this was harder as it is not clear weather creators have low beta or a high beta. From lines 576-579, it seems that data is the size of the edit. This is a little bit at odds with the definition of beta and x_i. Moreover, using this definition, I'm a bit confused about why the cost or effort for a single edit is defined as it is. It seems to me that the creators’ effort for creating the same amount of information would be lower than curators, in other words, it seems to me that the reverse of what the authors propose makes more sense. Overall it seems to me that the authors should think about the concepts of beta and x a bit more, as they currently seem inconsistent. As a suggestion, I wonder if creation versus curation can be considered just in terms of heterogeneity for the amount of content created, that is, x_i, removing the need for beta.

Response:

As we explain above, creators of content have a relatively large β**_i_**. We are no longer discussing the size of an edit; rather the definition of β**_i_** is based on the typical change that one makes to a sentence.

We define effort as a linear function of the amount of content changed. Hence, when considering a given number of sentence owned, a creator that comes to own a sentence by originating it typically expands more effort, when compared to a curator that could own a sentence by only changing part of it.

Once again, we thank the reviewer for these comments, which have pushed us to rethink the details of the proposed conceptualization.

**R2.03**: It is not clear what f_i is when it is introduced first. Moreover, rather than identifying this cost as being applicable only for the cooperative contributors in line 493, it seems to me that the authors should represent this as being higher for cooperative contributors. This stems from the fact that this cost depends on w in a continuous manner. Additionally this cost does not seem to be impacting any of the results. I believe it would be nice if the authors explain and clarify this in the paper.

Response: f_i_ represents the cooperative work that happens outside the main article page, for example in the TalkPage or other auxiliary Wikipedia namespaces. As the reviewer mentioned, f_i_ is indeed larger for cooperative contributors. Hence, the cost of cooperation, f_i_, is weighted by the contributor’s cooperative/competitive orientation, f_i_ * (1-w_i_). In other words, if w_i_ is closer to 1 (competitive contributor), then this cost is smaller; whereas if w_i_ is closer to 0 (cooperative contributor), then the cost associated with cooperative works is larger. We have revised the text to clarify this.

As the reviewer pointed out, given that the term f_i_ is independent of the number of sentences owned within the focal article (i.e. the decision variable, x_i_), it does not impact our results. Namely, the cost of cooperation on auxiliary pages is only added for completeness. We hope that the revised manuscript makes this point clear.

**R2.04**: The same idea with the continuous w_i applies for the governance tax when discussing its effect.

Response: Thank you for this point. Indeed, w_i_, too, is a continuous variable, and thus the associated costs larger/smaller for competitive/cooperative contributors.

The revised manuscript includes a detailed explanation of all the costs involved in our analysis. Just like the reviewer pointed out, the cost to overcome neutrality enforcement, t, is higher for a contributor who wishes to change content such that it aligns with her perspective (i.e. a competitive orientation). Hence, this cost is modelled as t * w_i_ * x_i_.

**R2.05**: I Also made this point in the last round about the convex cost function. Linear is not convex when talking about cost. The paper you refer to is in the context of linear programming, not game theory, where a convex cost function is different from linear. I let the AE decide whether it is necessary to actually try a convex cost function. It doesn’t seem to me that this would impact the main results. If you decide to leave out the convex cost function you can just focus on what is in the paper, but there's no need to mention convex cost function and it being outside of the scope of this paper.

Response: The reviewer makes an important point. Indeed, a general convex cost function greatly complicates the analysis, and hence we left it outside the scope of the current study.

To avoid any misleading inferences, the revised manuscript no longer discusses a convex cost function.

**R2.06**: I believe that even if the findings are not used as intermediate findings to prove further results (which I think does take place going from equation 4 to 6), it is still useful to provide the results more formally. Perhaps you can use a theorem instead for some of the results, for example those coming from equation 4, the optimal amount of content created, and equation 6, fractional content ownership. Looking at some similar papers that consider cooperative game theory might also help you better represent the equilibrium results.

Response: We have given consideration to this suggestion. We now formulated all results that can be deduced analytically, as theorems and retained all intuitive inferences as bullet points.

**R2.07**: When explaining the impact of t on equation 4 starting in line 563, I don't see a second order quadratic factor in the denominator for t. Perhaps comparative statics might be a better way to show this.

Response: The second order quadratic factor of t follows by expanding the denominator term . The denominator is a square of a binomial term, if it is perceived purely as a function of t and when treating all other variables as constants.

We did not expand on this point, as analyzing it would be extremely long and complex and would not provide any valuable insights to the Nash equilibrium.

**R2.08**: I found the results pertaining to w and beta to be interesting. I'm wondering if there is a better way to show this by considering the two dimensions of user heterogeneity. In other words the authors can draw a plot with these two dimensions and then characterize the different types of contributors within that two-dimensional space to better demonstrate their results.

Response: The point is well taken.

We empirically analyzed the correlation between these two variables, and found that the Pearson correlation coefficient between β_i_ and w_i_ is 0.167. This suggests that the two variables are largely independent.

As per the reviewer’s suggestion, we also plotted between β_i_ against w_i_, as shown below. However, we find that the plot is not very useful, as it is difficult to draw from the plot any conclusions.

Please note that since the number of points in the plot is large (over 100,000), a sequence of adjacent points appears like a line; this is only a visual error.

The revised manuscript mentions the correlation between β_i_ and w_i_; however, since this plot adds little insight, we opted to leave it out of the manuscript.

**R2.09**: Is it really the case that the type of users has no correlation with the amount of edits? Do privileged members contribute the same amount as the other users? Even though the authors provide some literature on this it would be nice to see some actual evidence. Even if the there is some correlation between these, I believe that the results would continue to hold, but they just need to discuss this further.

Response: Our analytical results indicate that the number of sentences owned by a contributor (x_i_) hinges on both w_i_ and β_i_. Naturally, it is expected that x_i_ correlates with these two variables.

We have now ran the empirical analyses. In our dataset, the correlation between x_i_ and w_i_ is 0.678 (i.e. contributors that have a competitive orientation tend to own more sentences). The correlation between x_i_ and β_i_ is -0.571 (curators tend to own more sentences).

**R2.10**: I believe that the piecewise nature of plot in figure 3 need additional explanation. What are the jumps that happen in these plots at around indexes 6, 8 and 9?

Response: We understand the reviewer’s concern on raising this point.

Normally, when using continuous variables, an analytical result would yield a smooth plot. However, the plots presented in Figure 3 are based on our empirical analysis, where variables are approximated based on Wikipedian’s data logs. Given that some of our proxies are ordinal (e.g. the estimate of w_i_, which is based on the contributor’s role within the community), the values for c_i_* show discontinuous behavior.

**R2.11** The non-human editors need additional information. What are the implications of this? This is a bit out of the blue.

Response:

Any study of Wikipedia that employs empirical data should consider the definition of “a contributor”. For example, Wikipedia logs associate any activity (or transaction) with the user’s IP address, and most scholarly investigations of Wikipedia consider unique IP addresses as distinct contributors. This is an approximation, as it is possible that multiple contributors have used a single computer (and thus a single IP address). Similarly, it is possible for a contributor to edit Wikipedia through more than one computer, thus use multiple IP addresses.

Along the same lines, the definition of “a contributor” should also consider whether or not to include software robots (in short, bots) in the analysis. Bots in Wikipedia are logged in the system as any other user (i.e. they have a user name). An empirical study of Wikipedia makes the decision re the inclusion/exclusion of bots based on the nature of the study. In our particular study, we thought that it is more appropriate to exclude bots, since it would be awkward to assign software scripts with intentions, such as cooperative/competitive orientation and the desire to own content.

**R2.12**: It wasn’t clear to me how the level of governance t was measured in the empirical part.

Response: The empirical data only measures the “follower” part of the leader-follower game. The results regarding governance, t, are based only on the analytical analysis. Given that the level of governance is assumed to be equal across Wikipedia (i.e. the same for all articles), there is no variation between articles and thus no way to analyze this aspect empirically.

**R2.13**: In the introduction before getting to the results the definition of governance is a little bit vague. It would help if the authors can add some examples of what governance actually means.

Response: The reviewer’s point is well taken.

In the context of our investigation, governance refers to neutrality enforcement mechanisms, including tools - automated algorithms and manual procedures - to detect intentional manipulation of content, as well as procedures to deal with manipulators (Forte et al., 2009; Edwards, 2013). One noticeable example is a commercially-sponsored attempt to bias Wikipedia's content by using multiple user accounts (Golumbia, 2013; McKenzie et al., 2012; Richards, 2014).

This was already explained in Section 2.1. However, per the reviewer’s request, we now also briefly explain this in the Introduction section.
